# Role of the Reproductive Tract Microbiome in Infertility and Assisted Reproductive Technologies: A Comprehensive Review

**DOI:** 10.7759/cureus.114891

**Published:** 2026-08-20

**Authors:** Daniela Ruiz, Victoria Robinson, Mariam Doucoure, Amara R Cheema, Carolina Ramirez Cabal, Afrah Saleef Abdul Salam, Rimane Abi Younes, Weiai Song, Sarah Ayoub Ibrahim, Olushola Ariyo, Rajat Das Gupta

**Affiliations:** 1 School of Medicine, Universidad Autonoma de Guadalajara, Guadalajara, MEX; 2 Rowan-Virtua School of Osteopathic Medicine, Rowan University, Stratford, USA; 3 Medicine, Istanbul Medipol University, Istanbul, TUR; 4 Medicine, Saint James School of Medicine, Park Ridge, USA; 5 Medicine, City St George's, University of London, London, GBR; 6 Hamdard Institute of Medical Sciences and Research, Jamia Hamdard, New Delhi, IND; 7 Medicine, Lebanese American University, Beirut, LBN; 8 Medicine, University College London Medical School, London, GBR; 9 Medicine, Istinye University, Istanbul, TUR; 10 Obstetrics and Gynecology, North Middlesex University Hospital, London, GBR; 11 Medicine, Vanderbilt University Medical Center, Nashville, USA

**Keywords:** assisted reproductive technology, female reproductive tract, infertility, microbiota, reproductive sciences

## Abstract

The female reproductive tract microbiome has emerged as a potential factor associated with reproductive health, infertility, and outcomes of assisted reproductive technologies (ARTs). Growing interest in host-microbiome interactions has highlighted the potential roles of microbial communities in implantation, immune regulation, and endometrial receptivity.

This comprehensive narrative review summarizes current evidence on the reproductive tract microbiome and its relationship with infertility and ART outcomes. Relevant literature was identified from MEDLINE, Embase, and Web of Science from database inception through March 2026, with emphasis on observational, interventional, and mechanistic studies involving human participants or human-derived samples. The review examines microbial communities of the vagina, cervix, endometrium, and fallopian tubes and their associations with infertility, recurrent pregnancy loss, implantation failure, and ART outcomes. Proposed biological mechanisms, including alterations in epithelial barrier integrity, immune modulation, inflammatory signaling, and hormonal-microbial interactions, are also discussed. In addition, the review evaluates emerging microbiome-based diagnostic approaches and potential therapeutic strategies, including antibiotics, probiotics, micronutrient supplementation, and vaginal microbiota transplantation.

Current evidence suggests that the reproductive tract microbiome may be associated with reproductive health and fertility outcomes and represents a promising area of translational research. However, substantial methodological heterogeneity, limited high-quality interventional evidence, and the lack of standardized diagnostic criteria currently restrict clinical implementation. Well-designed prospective studies and randomized clinical trials are needed to clarify causal relationships, evaluate microbiome-targeted interventions, and determine whether microbiome-based approaches can improve infertility and ART care.

## Introduction and background

Infertility, defined as the inability to achieve pregnancy after 12 months of regular unprotected intercourse, is a major global health concern, affecting approximately one in six adults worldwide [1,2]. The burden has increased substantially over recent decades and is accompanied by considerable psychological, social, and financial consequences [2,3]. Assisted reproductive technology (ART), including procedures such as in vitro fertilization (IVF), intracytoplasmic sperm injection (ICSI), and embryo transfer, provides important treatment options; however, access remains unequal, and treatment success remains variable [1,2,4].

Increasing attention has focused on the female reproductive tract (FRT) microbiome as a potential contributor to reproductive health. The FRT microbiome comprises microbial communities inhabiting anatomical sites, including the vagina, cervix, endometrium, and fallopian tubes. These communities vary across anatomical sites and physiological states. In the vagina, *Lactobacillus*-dominant microbial communities are commonly associated with maintenance of an acidic environment and reproductive tract homeostasis, whereas reduced *Lactobacillus* abundance accompanied by increased microbial diversity may represent dysbiosis in particular clinical contexts [5]. Advances in molecular methods, particularly 16S rRNA sequencing and other next-generation sequencing approaches, have also challenged earlier assumptions regarding microbial communities within the upper reproductive tract and stimulated investigation of their potential involvement in implantation, immune regulation, and endometrial receptivity [5,6].

Emerging evidence has therefore examined whether alterations in reproductive tract microbial composition are associated with infertility, implantation failure, pregnancy loss, and ART outcomes. However, the evidence remains heterogeneous, and microbiome-based diagnostic and therapeutic approaches have not yet been established for routine clinical practice [5,6]. Therefore, this comprehensive narrative review examines the role of the reproductive tract microbiome in infertility and ART outcomes, with particular emphasis on microbial variation across reproductive tract sites, associations with reproductive outcomes, potential biological mechanisms, and emerging diagnostic and therapeutic strategies aimed at improving reproductive and ART outcomes.

## Review

Methods

This study was designed as a comprehensive narrative review. To identify relevant literature, we conducted a structured literature search of MEDLINE, Embase, and Web of Science from database inception through March 2026. The purpose of the search was to identify representative and clinically relevant evidence addressing the reproductive tract microbiome, infertility, and ART, rather than to generate an exhaustive dataset for quantitative evidence synthesis.

Search terms included combinations of “female reproductive tract microbiome,” “vaginal microbiome,” “endometrial microbiome,” “dysbiosis,” “Lactobacillus,” “infertility,” “recurrent implantation failure,” “in vitro fertilization,” and “assisted reproductive technology.” Observational and interventional human studies and relevant mechanistic studies using human-derived samples were considered. Systematic reviews and meta-analyses were also examined to identify relevant primary literature. Animal-only studies and studies focused exclusively on overt reproductive tract infections without microbiome profiling or fertility-related outcomes were excluded.

As this was a narrative review, formal systematic review procedures such as protocol registration, duplicate independent screening, PRISMA study selection reporting, and formal risk-of-bias assessment were not undertaken. Evidence was synthesized thematically according to reproductive tract site, reproductive outcomes, proposed biological mechanisms, diagnostic approaches, and therapeutic interventions. Greater emphasis was placed on studies with clearly described methodologies, clinically relevant reproductive outcomes such as implantation, clinical pregnancy, or live birth, and studies providing mechanistic insight into host-microbiome interactions. Given substantial heterogeneity in study populations, microbiome assessment methods, sequencing platforms, microbial classifications, and outcome definitions, no quantitative meta-analysis was undertaken.

The female reproductive tract microbiome

The human microbiome plays a significant role in overall health, with microbial communities engaging in mutualistic interactions with the host [7]. The term “microbiome,” introduced by Joshua Lederberg in 2001, refers to an ecological community of microorganisms inhabiting a specific environment, together with their genetic material and interactions with the host. Within the FRT, microbial communities form dynamic ecosystems across the vagina, cervix, endometrium, and fallopian tubes, with composition varying according to anatomical site and physiological conditions.

Vaginal Microbiota

Community state types: The vaginal microbiome is commonly classified into five community state types (CSTs) based on 16S rRNA gene sequencing and the predominance of *Lactobacillus* species. CST I is dominated by *Lactobacillus crispatus*, CST II by *Lactobacillus gasseri*, CST III by *Lactobacillus iners*, and CST V by *Lactobacillus jensenii*. In contrast, CST IV is characterized by greater microbial diversity and non-*Lactobacillus*-dominant (NLD) anaerobes, including Gardnerella vaginalis, Atopobium vaginae, Prevotella, Mobiluncus, and Megasphaera [8,9]. CST I and V are generally considered protective because they contribute to maintenance of a low vaginal pH and resistance to pathogen overgrowth, whereas CST IV is commonly associated with bacterial vaginosis and vaginal dysbiosis.

Lactobacillus-dominant and non-Lactobacillus-dominant profiles: *Lactobacillus* species contribute to vaginal homeostasis through maintenance of an acidic pH, inhibition of pathogen overgrowth, immunomodulation, and epithelial barrier protection [9-11]. Among these species, *L. crispatus* is particularly associated with a stable vaginal environment [12]. In contrast, NLD communities are characterized by reduced *Lactobacillus* abundance and increased representation of anaerobic organisms [13]. The reproductive implications of *Lactobacillus*-dominant and NLD profiles are discussed in subsequent sections.

Endometrial and Upper Reproductive Tract Microbiota

Cervical microbiota: The cervical canal represents a transitional environment between the vagina and uterus and contains both *Lactobacillus* and non-*Lactobacillus* species. Ascending vaginal microorganisms may colonize the cervix and influence local immune responses. Reduced *Lactobacillus* dominance and human papillomavirus (HPV) infection have been associated with cervical cancer [14,15], while alterations in cervical microbial composition may facilitate pathogen ascent and contribute to pelvic inflammatory disease and infertility. Population-level variation in reproductive tract microbial composition should also be considered when interpreting NLD profiles, as greater microbial diversity may not necessarily indicate pathological dysbiosis in all populations [16]. Emerging evidence further suggests that cervical microbial composition may be associated with ART outcomes, although its clinical utility remains uncertain [17].

Endometrial microbiota: The endometrial microbiome is influenced by hormonal, menstrual, and clinical factors and has increasingly been investigated in relation to implantation and fertility. Studies have identified both *Lactobacillus*-dominant and NLD endometrial profiles [18-21]. Altered endometrial microbial composition has also been reported in association with endometriosis, chronic endometritis, fibroids, and implantation disorders [18,22-24]. Observational studies have generally associated *Lactobacillus*-dominant endometrial profiles with more favorable implantation, pregnancy, and live-birth outcomes [25,26]. In contrast, NLD profiles, including communities enriched with organisms such as *Gardnerella*, *Prevotella*, and *Atopobium*, have been associated with poorer implantation and reproductive outcomes and with disorders such as chronic endometritis [27,28]. However, characterization of the endometrial microbiome remains challenging because the endometrium is a low-biomass environment that is particularly susceptible to contamination.

Fallopian tube and ovarian microbiota: The microbial communities of the fallopian tubes and ovaries remain less well characterized than those of the vagina and endometrium. Taxa identified in the upper reproductive tract include *Pseudomonas*, *Acinetobacter*, *Vagococcus*, and *Sphingobium*, with relatively limited *Lactobacillus* abundance [29,30]. Alterations in these microbial communities have been proposed to contribute to tubal pathology and infertility; however, evidence remains limited. Interpretation of these findings requires caution because low microbial biomass and potential contamination complicate characterization of a normal upper reproductive tract microbiome [31].

The major microbial profiles identified across anatomical sites of the FRT and their potential reproductive implications are summarized in Table 1.

**Table 1:** Female reproductive tract microbiome profiles and their potential reproductive implications. CST, community state type; BV, bacterial vaginosis; ART, assisted reproductive technology; IVF, in vitro fertilization. Associations summarized in this table are primarily observational and should not be interpreted as establishing causality.

Anatomical site/profile	Predominant or reported microorganisms	Main characteristics	Potential reproductive implications	References
Vagina – CST I	Lactobacillus crispatus	*Lactobacillus*-dominant; maintains low vaginal pH; inhibits pathogen overgrowth	Generally associated with vaginal homeostasis and a favorable reproductive environment	[8-12]
Vagina – CST II	Lactobacillus gasseri	*Lactobacillus*-dominant vaginal community	Generally considered part of a *Lactobacillus*-dominant vaginal environment	[8,9]
Vagina – CST III	Lactobacillus iners	*Lactobacillus*-dominant but may differ functionally from other *Lactobacillus*-dominant CSTs	Clinical implications may differ according to microbial context	[8,9]
Vagina – CST IV	*Gardnerella vaginalis*, *Atopobium vaginae*, *Prevotella*, *Mobiluncus*, *Megasphaera*	Greater microbial diversity; non-*Lactobacillus*-dominant; commonly associated with vaginal dysbiosis/BV	Associated with infertility and implantation failure in some studies; interpretation may vary across populations	[8,9,13,16]
Vagina – CST V	Lactobacillus jensenii	*Lactobacillus*-dominant; contributes to maintenance of low pH	Generally considered a protective vaginal community	[8,9]
Cervix	*Lactobacillus* and non-*Lactobacillus* species	Transitional microbial environment between vagina and uterus; may be influenced by ascending vaginal microbiota	Altered composition may facilitate pathogen ascent and has been investigated in relation to infertility and ART outcomes	[14,15,17,18]
Endometrium – *Lactobacillus*-dominant	Predominantly *Lactobacillus spp.*	Lower microbial diversity with *Lactobacillus* predominance	Associated in observational studies with more favorable implantation, pregnancy, and live-birth outcomes	[18,25,26]
Endometrium – non-*Lactobacillus*-dominant	*Gardnerella*, *Prevotella*, *Atopobium*, and other taxa	Reduced *Lactobacillus* abundance and increased representation of other microorganisms	Associated with reduced implantation and IVF success and with chronic endometritis and implantation disorders	[18-28]
Fallopian tubes/ovaries	*Pseudomonas*, *Acinetobacter*, *Vagococcus*, *Sphingobium*; limited *Lactobacillus*	Low-biomass microbial environment; normal composition remains uncertain	Dysbiosis may be associated with tubal pathology and infertility; interpretation is limited by contamination concerns	[29-31]

Determinants of Microbial Composition Across the FRT

Hormonal and life-course influences: Estrogen and progesterone influence glycogen availability, epithelial function, and microbial composition throughout the reproductive tract [32]. During reproductive age, estrogen-driven glycogen production promotes *Lactobacillus* growth [33]. Pregnancy is generally associated with increased *Lactobacillus* abundance and reduced microbial diversity [31]. Conversely, declining estrogen during perimenopause and menopause is associated with reduced glycogen availability, higher vaginal pH, decreased *Lactobacillus* abundance, and increased prevalence of organisms such as *Atopobium*, *Streptococcus*, and *Prevotella* [34-36].

Other physiological and environmental exposures, including sexual activity, antibiotic use, pregnancy-related hormonal changes, and reproductive factors, may also alter microbial composition [37-39].

Geographic and population variation: The composition of the reproductive tract microbiome varies across populations and geographic settings and may reflect cultural, dietary, environmental, socioeconomic, and other extrinsic factors [37]. Ravel et al. reported differences in the prevalence of vaginal CSTs across racial and ethnic groups, including a higher prevalence of CST IV among Black and Hispanic females [9]. Importantly, greater microbial diversity may not necessarily represent pathological dysbiosis in all populations. Geographic and population-specific variation should therefore be considered when interpreting reproductive microbiome profiles [9,16].

Microbiome and reproductive outcomes

Available evidence suggests that variation in reproductive tract microbial composition is associated with natural fertility and outcomes following ART. However, most available evidence is observational, definitions of microbial dominance differ across studies, and associations should not be interpreted as establishing causality.

Natural Fertility

The FRT microbiome may contribute to the local conditions required for conception and maintenance of early pregnancy [40,41]. Both vaginal and endometrial microbial composition have therefore been investigated as potential correlates of reproductive health. Several studies have reported associations between *Lactobacillus*-dominant profiles and favorable reproductive outcomes, whereas altered microbial composition has been associated with subfertility [12,25,28,42,43]. Nevertheless, differences in sampling methods, microbial classification thresholds, and study populations limit direct comparisons across studies.

Assisted Reproductive Technology Outcomes

Microbial composition has also been investigated as a potential predictor of ART outcomes. NLD endometrial profiles have been associated with lower implantation, clinical pregnancy, and live birth rates in several studies [40,44-46]. Similarly, women with *Lactobacillus*-dominant vaginal microbiota have demonstrated more favorable IVF and frozen embryo transfer outcomes than women with dysbiotic profiles in some studies [41].

Emerging evidence also suggests that cervical microbial composition may be relevant to ART outcomes. Higher abundances of organisms including *Lactobacillus iners*, *Atopobium vaginae*, *Anaerococcus*, and certain *Firmicutes* have been associated with less favorable outcomes [17,18]. However, heterogeneity in microbiome assessment and outcome definitions currently limits the clinical utility of these associations.

Recurrent Implantation Failure and Chronic Endometritis

Reproductive tract dysbiosis has been investigated in women experiencing recurrent implantation failure (RIF) and chronic endometritis. These conditions have frequently been characterized by reduced *Lactobacillus* abundance and increased representation of anaerobic and opportunistic bacteria, including *Gardnerella*, *Prevotella*, *Atopobium*, and *Megasphaera* [18,28,30].

Women with chronic endometritis may exhibit substantial depletion of *Lactobacillus* together with increased abundance of *Actinobacteria*, *Gardnerella*, *Prevotella*, *Atopobium*, *Streptococcus*, and *Enterococcus* [27]. These observations support a possible relationship between altered microbial communities, endometrial inflammation, and implantation failure, although the direction and causal nature of these relationships remain uncertain [18,43].

Mechanistic pathways linking the microbiome to implantation and ART outcomes

Observed associations between reproductive tract microbial composition and fertility have prompted investigation of several potential biological mechanisms. These include modulation of local immune responses, disruption of endometrial receptivity and epithelial integrity, interactions with reproductive hormones, and systemic effects mediated through the gut-reproductive axis.

Immune Modulation and Inflammatory Signaling

Successful implantation requires a carefully regulated balance between immune tolerance toward the semi-allogeneic embryo and protection against infection. Microbial communities within the FRT may influence this balance through interactions with local immune cells and cytokine signaling [18,30].

*Lactobacillus* species may contribute to immune homeostasis by influencing T-helper cell responses, short-chain fatty acid production, and pro-inflammatory cytokine signaling [47]. Conversely, altered microbial communities may stimulate pattern-recognition pathways, including toll-like receptors, and promote persistent inflammatory signaling [48]. Such inflammation provides a plausible biological pathway linking dysbiosis with impaired implantation.

Endometrial Receptivity and Epithelial Integrity

Endometrial receptivity refers to the period during which the endometrium becomes capable of supporting blastocyst attachment and implantation [49]. Disruption of synchronization between embryonic development and endometrial receptivity may contribute to implantation failure and early pregnancy loss [49,50].

Moreno et al. reported that the endometrial microbiota remained relatively stable between pre-receptive and receptive phases; however, NLD microbial composition was associated with poorer implantation outcomes even in an endometrium classified as hormonally receptive [18]. These observations suggest that microbial composition may interact with hormonal and immunological determinants of endometrial receptivity.

Hormonal-Microbial Interactions

Reproductive hormones and microbial communities have bidirectional relationships. Estrogen and progesterone influence epithelial physiology and microbial composition, while microbiome-associated signaling may modify inflammatory responses. Elevated estrogen and progesterone during pregnancy have been associated with reductions in inflammatory mediators, including nitric oxide, tumor necrosis factor-alpha, and interferon-gamma [51,52]. Progesterone may also promote *Lactobacillus*-dominant microbial profiles and downregulate toll-like receptor 4 expression in cervicovaginal epithelial cells [53].

Microbe-associated molecular patterns may further modulate toll-like receptor signaling and inflammatory responses in epithelial cells [47]. These interactions illustrate how hormonal and microbial pathways may jointly influence reproductive tract homeostasis.

Gut-Reproductive Axis

Microbial influences on reproductive health may extend beyond the reproductive tract. The gut microbiome contributes to systemic estrogen metabolism through the estrobolome and may thereby influence hormonal and inflammatory pathways relevant to reproductive function [54]. Alterations in the gut microbiome have been associated with reproductive conditions such as endometriosis and polyendocrine metabolic ovarian syndrome (PMOS), formerly known as polycystic ovary syndrome (PCOS) [30,42,43,54]. Bariatric surgery, which produces substantial changes in metabolic and gut microbial profiles, has been associated with improved fertility among some women with PMOS, although findings remain inconsistent [55-58]. The clinical significance of the gut-reproductive axis therefore requires further investigation.

Clinical translation and emerging interventions

Although reproductive microbiome research has generated potential diagnostic and therapeutic applications, the clinical utility of most approaches remains uncertain. It is therefore important to distinguish currently available diagnostic technologies from experimental microbiome-directed interventions. Current and emerging diagnostic and microbiome-targeted approaches relevant to infertility and ART, along with their principal findings, limitations, and clinical status, are summarized in Table 2 [59-97].

**Table 2:** Diagnostic and microbiome-targeted approaches in infertility and assisted reproductive technology. ART, assisted reproductive technology; ERA, endometrial receptivity analysis; rsERT, RNA-seq-based endometrial receptivity test; qPCR, quantitative polymerase chain reaction; RCT, randomized controlled trial; BV, bacterial vaginosis; VMT, vaginal microbiota transplantation.

Approach	Purpose/rationale	Evidence summarized in the review	Principal findings	Key limitations/clinical status	References
ERA/rsERT	Identify the individualized window of implantation and guide personalized embryo transfer	Observational studies and meta-analyses	Some studies reported improved implantation or reduced pregnancy loss, whereas others found no significant benefit over standard embryo transfer	Evidence inconsistent; larger RCTs needed before routine implementation	[59-67]
Culture-based microbiome assessment	Identify cultivable microorganisms and permit antimicrobial susceptibility testing	Established microbiological method	Cost-effective and permits phenotypic characterization	Many organisms cannot be readily cultured; time-consuming; operator- and protocol-dependent	[68-71]
qPCR	Targeted detection and quantification of microorganisms	Molecular diagnostic approach	Allows sensitive quantification of predefined microbial targets	Restricted to organisms targeted by selected primers	[68,72-74]
16S rRNA sequencing	Characterize bacterial community composition	Widely used molecular profiling method	Enables broader characterization of bacterial communities than conventional culture	Limited taxonomic/functional resolution; methodological heterogeneity and contamination concerns	[68,72,74]
Shotgun metagenomics/whole-genome sequencing	Provide higher-resolution, untargeted genomic characterization	Emerging molecular approaches	Can provide broader taxonomic and functional information	Cost, analytical complexity, standardization, and interpretation currently limit routine use	[73,75]
Metatranscriptomics, metaproteomics, and metabolomics	Characterize microbial activity and functional interactions	Emerging multi-omics approaches	May provide functional information beyond microbial composition alone	Limited clinical validation and lack of standardized interpretation	[72]
Targeted antibiotic therapy	Treat chronic endometritis or confirmed pathogenic overgrowth	Interventional/clinical studies	May improve reproductive outcomes in selected patients	Broad-spectrum treatment may disrupt beneficial *Lactobacillus* populations; should not be used indiscriminately	[17,76]
Probiotics	Restore or support *Lactobacillus*-dominant microbial communities	RCTs, observational studies, and meta-analyses	Some studies report improved microbial parameters; evidence for improved pregnancy outcomes remains inconsistent	Effects vary by strain, formulation, baseline microbiota, and route; meta-analysis showed no statistically significant improvement in clinical pregnancy	[77-87,97]
Vitamin D and probiotics	Modulate endometrial and inflammatory pathways alongside microbial restoration	Small studies/interventional evidence	Some studies reported improvements in viable pregnancy and inflammatory or implantation-related markers	Limited evidence; small studies and inconsistent control of confounding; larger trials needed	[87-90]
Antibiotic and probiotic approaches	Reduce pathogenic organisms followed by restoration of *Lactobacillus*-dominant communities	Preliminary studies	May improve vaginal microbial composition and lower vaginal pH	Optimal treatment sequence, strains, and long-term colonization remain uncertain	[17,18]
Vaginal microbiota transplantation (VMT)	Restore vaginal eubiosis through transfer of microbiota from screened healthy donors	Pilot study and case report	Pilot study reported prolonged BV remission in 4/5 women; a case report described microbiome restoration followed by term pregnancy	Experimental; reproductive efficacy unproven; donor screening, safety, ethical oversight, and long-term stability remain concerns	[91-95]

Diagnostic and Receptivity Assessment

Endometrial receptivity is regulated by estrogen and progesterone and occurs during a relatively brief window of implantation [59-61]. Endometrial receptivity analysis (ERA) and the RNA-seq-based endometrial receptivity test (rsERT) have been developed to characterize this window and guide personalized embryo transfer.

Some studies have reported improved implantation or reduced pregnancy loss with receptivity-guided personalized embryo transfer [62,63], whereas others have demonstrated no significant benefit compared with standard embryo transfer approaches [64-66]. Current evidence therefore remains insufficient to support universal implementation, and larger randomized trials are required [67].

Microbiome Profiling

Microbiome profiling approaches range from conventional culture to molecular and multi-omics techniques. Culture-based methods permit organism identification and antimicrobial susceptibility testing but are limited because many microorganisms cannot be readily cultured and results may be affected by growth conditions and operator-dependent procedures [68-71].

Molecular approaches, including qPCR, 16S rRNA amplicon sequencing, shotgun metagenomics, and whole-genome sequencing, permit more comprehensive characterization of microbial communities [68,72-75]. Emerging approaches such as metatranscriptomics, metaproteomics, and metabolomics may additionally provide information regarding microbial function and host-microbiome interactions [72]. However, lack of standardized sampling, sequencing, analytical, and interpretive criteria currently limits routine clinical application.

Microbiome-targeted therapeutic interventions

Antibiotic Therapy

Targeted antimicrobial treatment may be appropriate in selected patients with chronic endometritis or confirmed pathogenic overgrowth. Some studies suggest that treatment can improve reproductive outcomes among appropriately selected women [76]. However, indiscriminate broad-spectrum antibiotic use may disrupt beneficial Lactobacillus populations and potentially worsen microbial dysbiosis [17]. Antibiotic treatment should therefore be distinguished from generalized attempts to manipulate the reproductive microbiome.

Probiotics and Microbial Modulation

Probiotic interventions have primarily focused on restoration of *Lactobacillus* species. However, evidence regarding reproductive outcomes remains inconsistent. Meta-analytic evidence suggests only modest, statistically non-significant improvement in clinical pregnancy among ART patients receiving probiotics (RR: 1.19; p = 0.07) [77].

Individual studies have reported improvements in vaginal pH, *Lactobacillus* abundance, *Gardnerella vaginalis* abundance, and Nugent scores following oral or vaginal probiotic administration [78-80]. However, other studies have found no improvement in pregnancy outcomes among IVF or RIF populations [81-85]. Probiotic efficacy may depend on the specific strain, formulation, route of administration, baseline microbiota, and capacity for stable colonization [81,86,87].

Probiotics have also been studied in combination with vitamin D. Some studies suggest potential improvements in viable pregnancy and inflammatory markers [87-90], but much of the evidence derives from relatively small studies with methodological limitations. Larger randomized trials are required before these approaches can be recommended routinely.

Combination Approaches

Combination strategies involving targeted antibiotic therapy, followed by probiotic supplementation, have been investigated as a means of restoring *Lactobacillus*-dominant microbial communities. Preliminary evidence suggests potential improvement in vaginal microbial composition and pH [17]. Future approaches may need to prioritize stable colonization by beneficial species such as *L. crispatus* rather than simply increasing total bacterial abundance [18].

Emerging therapies and future clinical applications

Vaginal Microbiota Transplantation

Vaginal microbiota transplantation (VMT) is an experimental strategy involving transfer of vaginal microbiota from a healthy donor to a recipient with dysbiosis [91,92]. In a small pilot study, four of five women with recurrent bacterial vaginosis achieved prolonged remission following antibiotic pretreatment and VMT [93,94]. A case report also described microbiome restoration and subsequent term pregnancy following VMT in a woman with recurrent pregnancy loss and *Gardnerella*-dominant dysbiosis [94].

Despite these findings, evidence supporting reproductive benefits remains extremely limited, and controlled trials are lacking [92]. Donor screening, pathogen transmission, ethical oversight, and long-term microbiome stability are important considerations [92,95]. VMT should therefore currently be regarded as an experimental rather than established fertility treatment.

Future Clinical Applications

Future microbiome-directed reproductive care may involve individualized approaches integrating microbial composition with hormonal, immunological, and clinical characteristics. However, evidence supporting routine microbiome profiling or microbiome-targeted treatment remains insufficient. Clinical implementation will require validated microbial thresholds, standardized testing procedures, prospective evidence of predictive utility, and randomized trials demonstrating improvement in clinically meaningful outcomes such as live birth.

Limitations of current evidence and future research priorities

Although accumulating evidence suggests an association between the reproductive tract microbiome and fertility and ART outcomes, several methodological and conceptual limitations preclude definitive conclusions.

First, the evidence base remains predominantly observational, including cross-sectional and retrospective cohort studies [66,86]. Consequently, associations between microbial composition and reproductive outcomes cannot establish causality. Reverse causation and residual confounding remain important concerns [38,39,96,97].

Second, substantial methodological heterogeneity exists in specimen collection, DNA extraction, sequencing platforms, microbial classification, bioinformatic pipelines, and contamination control procedures [68,74]. These limitations are particularly important for low-biomass environments such as the endometrium [70,71].

Third, there is no universally accepted definition of *Lactobacillus* dominance or reproductive tract dysbiosis. Thresholds vary across studies, and individual *Lactobacillus* species may have different functional implications despite frequently being grouped together [20,96-98].

Fourth, reproductive outcomes are inconsistently defined across studies. Reported endpoints include biochemical pregnancy, implantation, clinical pregnancy, ongoing pregnancy, and live birth [18]. Greater use of standardized outcomes, particularly live birth, would improve comparability and clinical interpretation [67].

Fifth, high-quality interventional evidence remains limited. Randomized controlled trials evaluating antibiotics, probiotics, and other microbiome-targeted interventions with live birth as the primary outcome are scarce [86]. Evidence regarding emerging interventions such as VMT remains particularly preliminary [92,95].

Sixth, most studies focus predominantly on bacterial communities assessed using 16S rRNA sequencing. The potential contributions of viruses, fungi, microbial metabolites, and microbial functional activity remain comparatively underexplored. Metagenomic, metabolomic, transcriptomic, and other multi-omics approaches may provide a more comprehensive understanding of host-microbiome interactions [12,99].

Seventh, the lack of standardized global protocols remains a major barrier to translation. Harmonization of sampling procedures, contamination controls, sequencing methods, analytical approaches, and reporting standards is needed, particularly for low-biomass reproductive tract sites [98].

Finally, reproductive microbiome research remains geographically concentrated in high-income settings [100]. Greater representation of populations from low- and middle-income countries is needed because geographic, dietary, environmental, and socioeconomic factors may influence microbial composition and limit generalizability [101-103].

Future research should prioritize prospective longitudinal studies examining temporal changes in the reproductive microbiome and adequately powered randomized controlled trials evaluating microbiome-targeted interventions with live birth as a primary endpoint [67]. Standardization of sampling, sequencing, microbial definitions, and reproductive outcomes will be critical [63]. Integration of microbiome data with hormonal, immunological, genetic, and clinical information may ultimately improve understanding of host-microbiome interactions and inform appropriately validated precision reproductive approaches [12].

## Conclusions

The reproductive tract microbiome has emerged as a potential contributor to female fertility and ART outcomes. Observational studies suggest associations between vaginal and endometrial microbial composition and implantation, pregnancy, and live birth outcomes, while mechanistic studies support potential interactions with immune regulation, epithelial integrity, endometrial receptivity, and hormonal signaling. However, the available evidence remains limited by observational study designs, methodological heterogeneity, inconsistent definitions of dysbiosis and reproductive outcomes, and a lack of adequately powered randomized controlled trials. Consequently, the observed relationships should not be interpreted as establishing causality.

At present, microbiome profiling and microbiome-targeted interventions remain investigational in reproductive medicine. Prospective studies and randomized clinical trials using standardized methods and clinically meaningful endpoints, particularly live birth, are required before these approaches can be incorporated into routine infertility and ART care.
